# Dramatic Potentiation of the Antiviral Activity of HIV Antibodies by Cholesterol Conjugation*

**DOI:** 10.1074/jbc.M114.591826

**Published:** 2014-10-23

**Authors:** Krzysztof Lacek, Richard A. Urbanowicz, Fulvia Troise, Claudia De Lorenzo, Valeria Severino, Antimo Di Maro, Alexander W. Tarr, Francesca Ferrara, Alexander Ploss, Nigel Temperton, Jonathan K. Ball, Alfredo Nicosia, Riccardo Cortese, Antonello Pessi

**Affiliations:** From ‡ Ceinge Biotecnologie Avanzate S.C.R.L., Via Gaetano Salvatore 486, 80145 Napoli (NA), Italy,; the § Laboratory of Virus Molecular Biology, University of Gdansk, 80-822 Gdansk, Poland,; the ¶ School of Life Sciences and; ‖ Nottingham Digestive Diseases Centre Biomedical Research Unit, University of Nottingham, Queen's Medical Centre, Nottingham NG7 2UH, United Kingdom,; the ** Department of Molecular Medicine and Medical Biotechnology, University of Naples Federico II, Via Pansini 5, 80131 Napoli (NA), Italy,; the ‡‡ Department of Environmental, Biological and Pharmaceutical Sciences and Technologies, Second University of Naples, Via Vivaldi 43, 81100 Caserta (CE), Italy,; the §§ Viral Pseudotype Unit, Infectious Diseases and Allergy group, School of Pharmacy, University of Kent, Kent ME4 4TB, United Kingdom,; the ¶¶ Department of Molecular Biology, Princeton University, Princeton, New Jersey 08544, and; ‖‖ JV Bio, Via Gaetano Salvatore 486, 80145 Napoli (NA), Italy

**Keywords:** Antibody Engineering, Cholesterol, Human Immunodeficiency Virus (HIV), Lipid Raft, Membrane Fusion, Molecular Evolution, Viral Immunology, Virus Entry

## Abstract

The broadly neutralizing antibodies HIV 2F5 and 4E10, which bind to overlapping epitopes in the membrane-proximal external region of the fusion protein gp41, have been proposed to use a two-step mechanism for neutralization; first, they bind and preconcentrate at the viral membrane through their long, hydrophobic CDRH3 loops, and second, they form a high affinity complex with the protein epitope. Accordingly, mutagenesis of the CDRH3 can abolish their neutralizing activity, with no change in the affinity for the peptide epitope. We show here that we can mimic this mechanism by conjugating a cholesterol group outside of the paratope of an antibody. Cholesterol-conjugated antibodies bind to lipid raft domains on the membrane, and because of this enrichment, they show increased antiviral potency. In particular, we find that cholesterol conjugation (i) rescues the antiviral activity of CDRH3-mutated 2F5, (ii) increases the antiviral activity of WT 2F5, (iii) potentiates the non-membrane-binding HIV antibody D5 10–100-fold (depending on the virus strain), and (iv) increases synergy between 2F5 and D5. Conjugation can be made at several positions, including variable and constant domains. Cholesterol conjugation therefore appears to be a general strategy to boost the potency of antiviral antibodies, and, because membrane affinity is engineered outside of the antibody paratope, it can complement affinity maturation strategies.

## Teaser

**Background:** Some HIV-neutralizing antibodies have an antigen-binding site with dual specificity, for the plasma membrane and a viral epitope.

**Results:** We engineered membrane affinity outside the antigen-binding site by conjugating cholesterol, with concomitantly increased antiviral potency.

**Conclusion:** A natural mechanism dependent on affinity maturation is mimicked by conjugation of a lipid.

**Significance:** This is a general strategy to boost the potency of antiviral antibodies.

## Introduction

The broadly neutralizing antibody (bnAb)^3^ 2F5 is perhaps the most extensively studied of all HIV antibodies. It binds to the membrane-proximal external region (MPER) of the fusion protein gp41 (1). The MPER (residues 662–683) lies at the base of the gp41 ectodomain, immediately proximal to the transmembrane domain; it is highly conserved and critical for viral fusion (2). Another bnAb, 4E10, binds to an overlapping epitope (3). A key feature of both antibodies is the presence of a relatively long, hydrophobic CDRH3 loop, most of which does not contact the peptide antigens in the crystal structures (3, 4) and yet is essential for the antiviral activity (5, 6). In particular, two independent studies have shown that mutagenesis of the hydrophobic residues at the tip of the CDRH3 or ablation of the apex of the same loop can completely abolish the neutralizing activity of 2F5 and 4E10 while inducing no change in the affinity for the peptide epitope (5–8). The same studies have shown that the CDRH3 residues promote binding of the antibodies to synthetic lipid bilayers (6, 7), including liposomes that mimic the lipid composition of the HIV-1 membrane (9).

These results have led to the proposal of a two-step mechanism for HIV neutralization by 2F5 (5, 6) and 4E10 (6). In step 1, the antibodies attach to the viral membrane through their long, hydrophobic CDRH3 loops. This weak reversible binding preconcentrates the antibody on the virion surface until binding of gp120 to CD4 and the chemokine coreceptor triggers the gp41 conformational change leading to formation of the prehairpin intermediate, with the fusion peptide inserted into the target cell membrane (10). As a consequence, during the relatively short window of opportunity (estimated to be about 15 min (11)) before fusion is completed, the local concentration of the antibody in the vicinity of the MPER epitope is much higher than the bulk aqueous concentration, driving formation of a high affinity complex (step 2), which prevents fusion from proceeding to completion.

An open question, with important practical implications, is whether an MPER bnAb simply needs to possess the two properties or if a very specific geometry of the combining site is required, as present in 2F5 and 4E10. Two recent studies seem to support the latter hypothesis, proposing that the CDRH3 loop of 2F5 participates in a membrane-embedded “extended paratope” (12, 13). A third study, however, questions this conclusion (13).

If the two-step hypothesis is true, one could imagine engineering membrane affinity outside of the antibody paratope, in this way making general the special mechanism of 2F5/4E10. To address this issue, we have sought a general way to increase the affinity of an antibody for lipid membranes without the need of a special sequence in its CDRH3.

A substantial body of evidence supports the notion that viral fusion occurs in confined areas of the interacting viral/host membranes (14). For HIV, the lipid composition of the viral membrane is strikingly different from that of the host cell membrane, being particularly enriched in cholesterol and sphingomyelin (9, 15–17). Cholesterol and sphingolipids are often laterally segregated in membrane microdomains or “lipid rafts” (9, 19). We have recently shown that the efficient targeting to lipid rafts can be achieved by conjugation of a cholesterol group and that this modification brings a dramatic increase in antiviral potency of peptide fusion inhibitors derived from the heptad repeat region of fusion proteins, including gp41 (20–25). Our findings have been independently confirmed (26). Conjugation to sphingosine has also been shown to potentiate antiviral activity (27). Importantly, cholesterol conjugation has been shown to efficiently localize the much larger green fluorescent protein to the membrane of living cells (28). Based on this, we asked if conjugation of cholesterol to a previously described CDRH3 mutant of 2F5, which is unable to neutralize HIV (5), could rescue its antiviral activity. We show here that this is the case and that cholesterol conjugation can also increase the antiviral activity of another HIV antibody, D5, which is devoid of membrane affinity.

## EXPERIMENTAL PROCEDURES

### 

#### 

##### Cell Lines

Human embryonic kidney 293 cells (HEK 293 ATCC CCL1573) were grown in Dulbecco's modified essential medium (DMEM; Invitrogen) supplemented with 10% FBS. HEK 293 EBNA cells (Invitrogen, catalogue no. R620-07) were grown in DMEM with 10% FBS, 1% nonessential amino acids (Invitrogen), and G418 (Invitrogen) at 0.25 mg/ml. The HEK 293T cell line was purchased from ECACC, and the HeLa TZM-bl cell line was obtained through the Programme EVA Centre for AIDS Reagents, National Institute for Biological Standards and Control (NIBSC). Both were grown in DMEM with 10% FBS and 1% nonessential amino acids. Monoclonal antibodies were produced in HEK 293 EBNA cells cultured in CDCHO medium (Invitrogen) supplemented with 100 μm hypoxanthine and 16 μm thymidine (Invitrogen).

##### Generation of HIV-1 Pseudovirus

Human embryonic kidney 293T/17 cells (ATCC: CRL-11268) (1.2 × 10^6^) were seeded in 10-cm^2^ dishes. After 24 h, cells were co-transfected with 2 μg each of a pNL4.3 construct lacking *env* and an *env*^+^ pcDNA3.1 D-TOPO expression vector using Lipofectamine 2000 (Invitrogen). 72 h post-transfection, culture supernatants containing pseudotype virus were filtered through 0.22-μm filters and stored at 4 °C.

##### Generation of Human Monoclonal Antibodies

All tested mAbs were generated by subcloning of synthetic heavy and light chain variable regions (GeneArt, Invitrogen) into two eukaryotic vectors for the expression of heavy (IgG1 isotype) and light chains, as described previously (29). These two plasmids were co-transfected into HEK 293 EBNA cells with Lipofectamine 2000 reagent (Invitrogen), and whole human IgG was purified from culture medium with Hi-Trap protein A columns (GE Healthcare).

##### Conjugation of Cholesterol to mAbs

**FIGURE 1.**
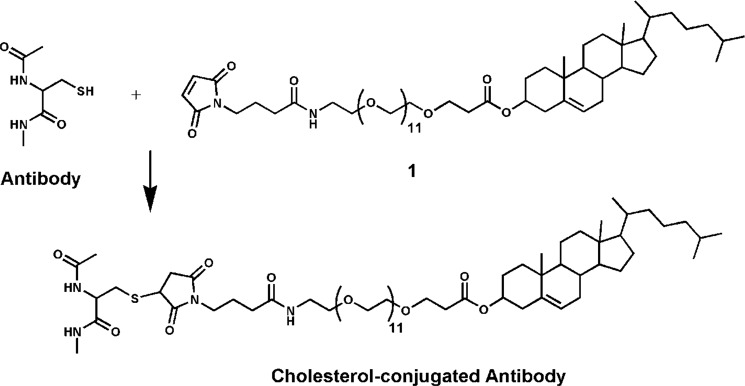
**Chemistry used for conjugation of cholesterol to an antibody.** Maleimido-PEG_12_-chol is conjugated to the unpaired cysteine residues introduced in the antibody sequence.

Cholesterol-PEG_12_-maleimide (compound **1** in Fig. 1) was obtained from Charnwood Molecular Ltd. (Loughborough, UK). To ensure reactivity of the free cysteines introduced in the mAb sequence, the antibodies were incubated for 2 h at 37 °C with a 200-fold molar excess of l-cysteine in triethylamine buffer (pH 8.0) in N_2_ atmosphere. Reduced mAbs were buffer-exchanged into conjugation buffer (10 mm sodium phosphate, 5 mm EDTA, pH 7.2) on PD-10 desalting columns (GE Healthcare). Next, octyl β-d-glucopyranoside (Sigma) was added to the mAbs solution to a final concentration of 40 mm, and antibodies were reacted with a 10-fold molar excess of cholesterol-PEG_12_-maleimide for 2 h at room temperature. mAbs were then bound to Protein A-Sepharose (Sigma) overnight at room temperature with constant rotation in a vertical rotator. Bound mAbs were washed four times with 20 resin volumes of 40 mm octyl β-d-glucopyranoside in conjugation buffer, followed by four washing steps with 20 resin volumes of plain conjugation buffer. mAbs were eluted from resin with 100 mm glycine-HCl (pH 2.7) and neutralized with the addition of 1 m Tris-HCl (pH 9.0) to a final concentration of 100 mm. Eluted mAbs were buffer-exchanged into PBS on PD-10 desalting columns (GE Healthcare). Conjugation efficiency was analyzed by 12% SDS-PAGE and mass spectrometry, and the results were in good agreement.

Conjugation efficiency varied with the reaction conditions. Although it was possible to fully derivatize the unpaired cysteine residue, driving conjugation to completion led to the appearance of SDS gel bands corresponding to incorporation of more than two maleimido-PEG_12_-chol molecules per antibody (confirmed by MS). Although maleimide-based reagents are selective for free thiols at neutral pH, reaction with primary amines has been documented at higher pH (30), and the p*K_a_* of individual lysine residues can be influenced by structural and environmental features (31).

Hence, it was decided to use the conditions reported above, which led to the target antibody with two cholesterol groups per molecule in the presence of residual unconjugated antibody. The percentage of conjugation varied from 50 to 70%.

**FIGURE 2.**
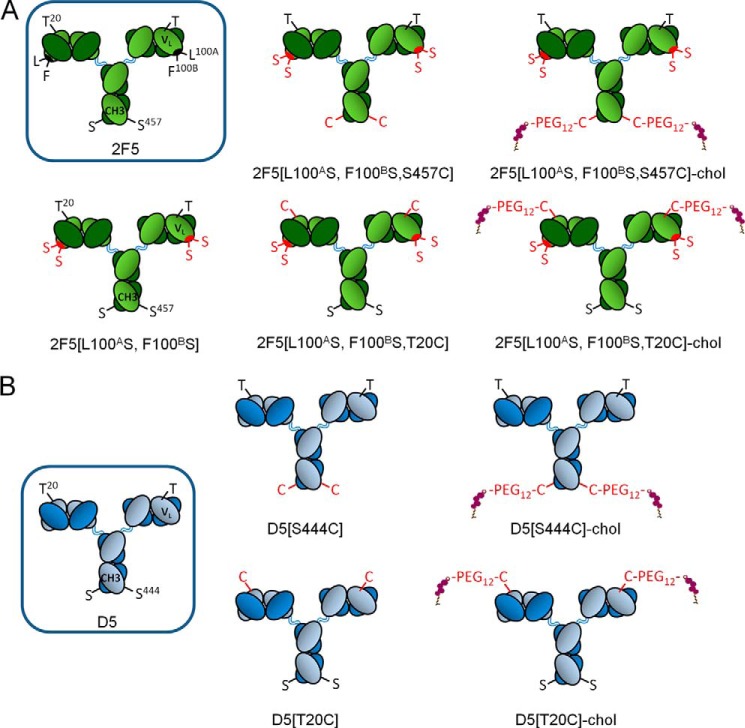
**Schematic representation of the cholesterol-conjugated antibodies.** The WT residues are shown in *black*, whereas the mutated residues are highlighted in *red*. Cholesterol is shown in *violet. A*, 2F5; *B*, D5.

For the experiments described here, the concentration of cholesterol-conjugated antibody was adjusted based on the SDS data. Because the difference in antiviral IC_50_ between the cholesterol-conjugated/unconjugated antibodies was always >10-fold and typically 100-fold, the presence of residual unconjugated antibody in the conjugated antibody sample was considered irrelevant for the interpretation of results. The analytical data for the antibodies described here are reported in Fig. 2.

##### Binding of HIV-1 mAbs to HEK 293 Cells

HEK 293 cells (7.5 × 10^5^) were incubated for 1 h at room temperature with HIV-1 mAbs in phosphate-buffered saline (PBS) containing 0.2% bovine serum albumin (BSA) and 10 mm HEPES (fluorescence-activated cell sorter (FACS) buffer). mAb binding was revealed by Fcγ-specific, allophycocyanin-conjugated goat antihuman IgG. As a control, isotype-matched human IgG1 was used. FACS acquisitions and analyses were performed with a FACSCalibur (BD Biosciences) and CellQuest software.

##### Treatment with Cholesterol-depleting Agents

HEK 293 cells were washed twice with serum-free DMEM and treated for 1 h at 37 °C with 10 mm methyl-β-cyclodextrin (Sigma) diluted in serum-free DMEM. After incubation, cells were washed three times with serum-free DMEM prior to flow cytometry (FACS) assays.

##### HIV-1 Infectivity and Neutralization Assays

HeLa TZM-bl cells (2 × 10^5^) were seeded in 96-well Optilux plate (BD Biosciences). After 24 h, pseudotype virus and 200 μl of medium were added and incubated at 37 °C in a humidified atmosphere containing 5% CO_2_, for 72 h. Medium was then removed, cells were lysed with 50 μl of cell lysis buffer (Promega), and 50 μl of luciferase substrate (Promega) was added. Luminescence was measured by using a BMG Labtech Optima plate reader. For neutralization assays, pseudotype virus was mixed with dilutions of mAbs, incubated for 1 h at 37 °C, and then added to TZM-bl cells plated into a 96-well Optilux plate containing 100 μl of medium, and the plates were incubated for 4 h before an additional 100 μl of medium was added. Cultures were incubated and read as for the infectivity assay. For cell binding assays, dilutions of mAbs were added to TZM-bl cells plated into a 96-well Optilux plate and incubated for 1 h at 37 °C. They were then washed with PBS to remove unbound antibody, and pseudotype virus was added. The plates were incubated for 4 h before an additional 100 μl of medium was added. Cultures were incubated and read as for the infectivity assay.

##### Combination Experiment

Synergistic, additive, or antagonistic interaction by two antibodies for virus neutralization was evaluated by the median effect analysis method, as described (32), using the CompuSyn software (ComboSyn Inc., Paramus, NJ). This approach takes the potency, shape, and slope of the dose-dependent neutralization curve of each antibody into account, both alone and in combination, at a constant ratio, to calculate a combination index (CI). A CI value of <1 indicates synergism, 1 indicates an additive effect, and >1 indicates antagonism. For each antibody, dose-dependent neutralization was initially measured to determine a concentration range. Neutralization values of serial 5-fold dilutions of each antibody alone and in combination were then measured in a range of concentrations above and below the IC_50_ values. The measured neutralization values were entered into the program as fractional effects in the range of 0.01 < fractional effect < 0.99 for each of the two antibodies and for both in combination. The software determines the linear correlation coefficient, *r*, of each curve to indicate the fit or conformity of the data with respect to the median effect method and calculates the CI values in relation to fractional effect values. The cultures were incubated and read as for the neutralization assay.

##### Mass Spectrometry

Prior to MALDI-TOF analysis, each antibody (0.3 mg/ml diluted in 50 mm ammonium bicarbonate) was reduced by the addition of 5 μl of 0.2 m dithiothreitol (DTT) for 30 min at 37 °C, followed by immediate cooling at 4 °C. MS analysis was performed on a MALDI-TOF micro MX instrument (Waters Co., Manchester, UK) equipped with a pulsed nitrogen laser (λ = 337 nm). The instrument source voltage was set to 12 kV. The pulse voltage was optimized at 1999 V, and the detector voltage was set to 5200 V. Measurements were performed in the mass range *m*/*z* 20,000–80,000, with a suppression mass gate set to *m*/*z* 3000 to prevent detector saturation from matrix cluster peaks and an extraction delay of 600 ns. Prior to acquisition of spectra, 1 μl of reduced antibody solution was mixed with 1 μl of saturated sinapinic acid matrix solution (10 mg/ml in acetonitrile/water containing 0.1% trifluoroacetic acid (1:1.5, v/v)). A droplet (1 μl) of the resulting mixture was placed on the mass spectrometer's sample target and dried at room temperature. After complete evaporation of the liquid, the sample was loaded into the mass spectrometer and analyzed in positive acquisition linear mode. External calibration was with a mixture of standard proteins (10 pmol/μl each of insulin, cytochrome *c*, trypsinogen, and albumin; Sigma). Mass accuracy near the nominal value (300 ppm) was achieved for each standard. All spectra were processed and analyzed using MassLynx version 4.1 software (Waters, Milford, MA).

##### ELISA

The peptide antigens for the ELISA experiments were prepared by standard Fmoc (*N*-(9-fluorenyl)methoxycarbonyl) solid-phase methods. Peptide sequences were as follows: P1728, Glu-Leu-Leu-Glu-Leu-Asp-Lys-Trp-Ala-Ser-Leu-Trp-Asn-Lys(*N*^ϵ^-biotin)-NH_2_; P2370, Ac-Ile-Glu-Lys-Lys-Ile-Glu-Ala-Ile-Glu-Lys-Lys-Ile-Glu-Ala-Ile-Glu-Lys-Lys-Ile-Glu-Ala-Ile-Glu-Lys-Leu-Leu-Gln-Leu-Thr-Val-Trp-Gly-Ile-Lys-Gln-Leu-Gln-Ala-Arg-Ile-Leu-NH_2_. For ELISA with the biotinylated HIV-1 antigen P1728, 96-well plates (Immunoplate Maxisorp, Nunc) were precoated with 0.25 μg/well streptavidin (Thermo Scientific) in coating buffer (50 mm NaHCO_3_, pH 9.6) overnight at 4 °C and washed four times with PBST buffer (0.05% Tween 20 in PBS). Plates were incubated for 2 h at room temperature with 1 μg/well P1728 in coating buffer, subsequently washed with PBST buffer, and blocked for 1 h at 37 °C with 250 μl/well of milk buffer (5% milk in PBST). Plates were incubated with various HIV-1 antibodies diluted in milk buffer overnight at 4 °C, washed, and incubated for 1 h at room temperature with anti-human IgG Fc-specific AP conjugate (Sigma) diluted 1:10,000 in milk buffer. Alkaline phosphatase was revealed by incubation with *p*-nitrophenyl phosphate substrate solution (Sigma). Results were expressed as the difference between *A*_405 nm_ and *A*_620 nm_, measured by an automated ELISA reader (Envision 2102 Multilabel reader, PerkinElmer Life Sciences). ELISA with the non-biotinylated peptide P2370 was performed with the same protocol, with the exception of the streptavidin precoating step.

##### Graphs and Statistics

Graphs were prepared using Prism version 5.0c or 6.0e (GraphPad Software Inc., La Jolla, CA) or SigmaPlot version 12.0 (Systat Software Inc., San Jose, CA).

## RESULTS

### 

#### 

##### Conjugation of Cholesterol to 2F5

A suitable method to generate an antibody-cholesterol conjugate is through thiol-maleimide chemistry, whereby a cysteine residue, either native or engineered, is used for conjugation on the side of the antibody, and a reactive maleimide group is used on the cholesterol moiety (33) (Fig. 1). A number of studies have shown that surface cysteines can be introduced in all domains of an antibody without interference with the inter- and intrachain disulfide bonds (33–36), giving us ample choice for the site of attachment. Because of the symmetrical nature of antibodies, two cholesterol groups are attached per antibody molecule.

**FIGURE 3.**
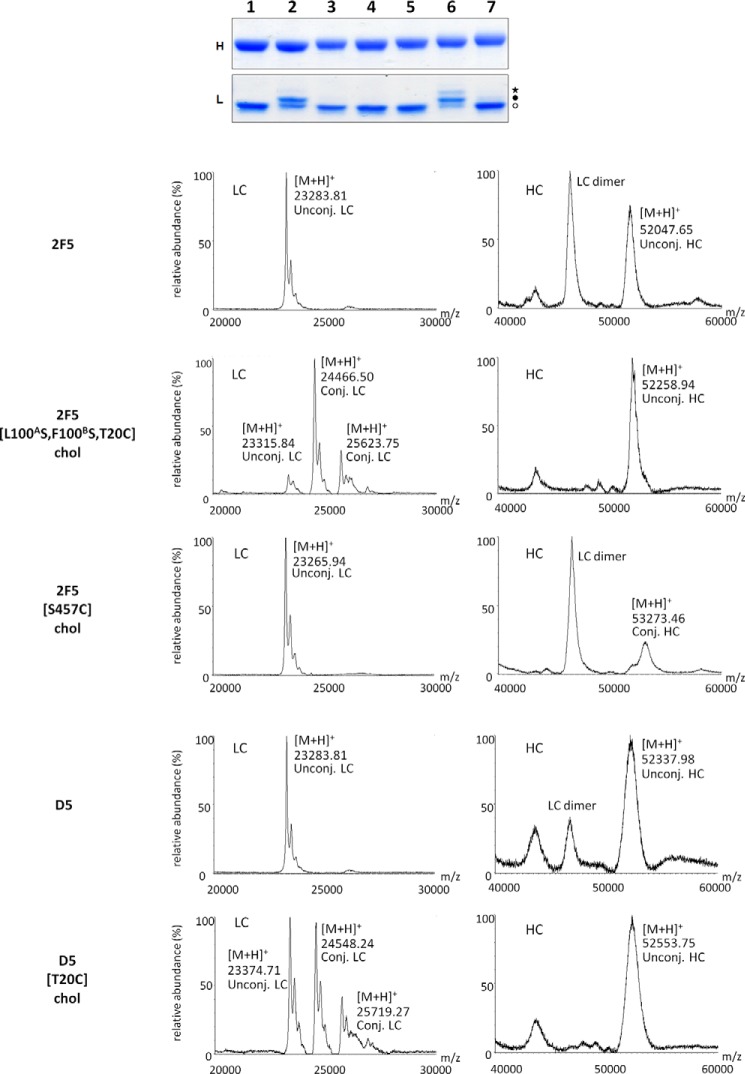
**Analytical characterization of cholesterol-conjugated antibodies.** *Top*, SDS-PAGE analysis. mAbs were separated by SDS-PAGE and stained with Coomassie Brilliant Blue. *Lane 1*, 2F5[L100^A^S,F100^B^S,T20C]; *lane 2*, 2F5[L100^A^S,F100^B^S,T20C]-chol; *lane 3*, 2F5[S457C]-chol; *lane 4*, 2F5[L100^A^S, F100^B^S, S457C]; *lane 5*, 2F5[L100^A^S, F100^B^S, S457C]-chol; *lane 6*, D5[T20C]-chol; *lane 7*, D5[S444C]-chol. The heavy and light chains of each mAb are shown in the *top* (*H*) and *bottom* (*L*) *panels*, respectively. Specific conjugation of the light chain resulted in the presence of higher molecular weight species (*filled circle*) in comparison with the unconjugated light chain (*open circle*). Additional species (*asterisk*) were observed if the light chain was also nonspecifically conjugated. *Bottom*, MALDI-TOF analysis of cholesterol-conjugated 2F5 and D5 antibodies.

**FIGURE 4.**
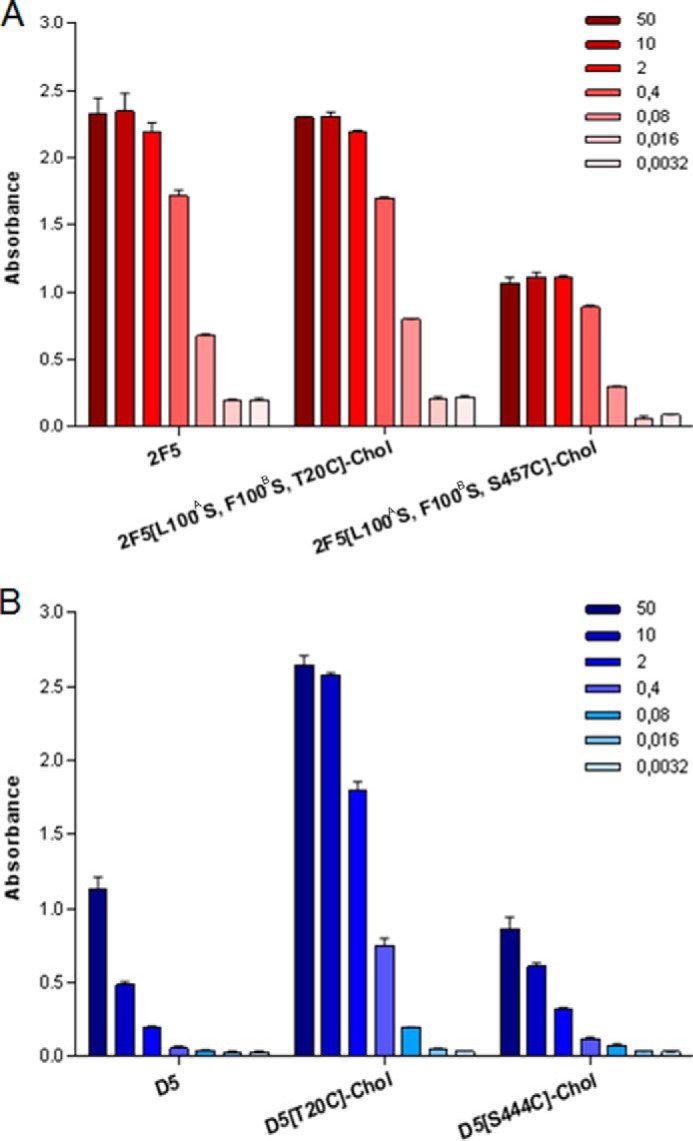
**Binding of 2F5 and D5 mAbs to gp41 peptides.** Binding of unconjugated and cholesterol-conjugated HIV-1 mAbs to the specific gp41 peptide antigen was analyzed by ELISA. 96-well plates were coated with either peptide P1728 (for 2F5 antibodies) or peptide P2370 (for D5 antibodies) and incubated with various concentrations of mAb 2F5 (*A*) or mAb D5 (*B*). Values on the *vertical axis* are absorbance measured at 405 nm. mAb concentration is shown on the *right* (in nm). The experiments were performed in duplicate, and the *error bars* represent S.D.

**FIGURE 5.**
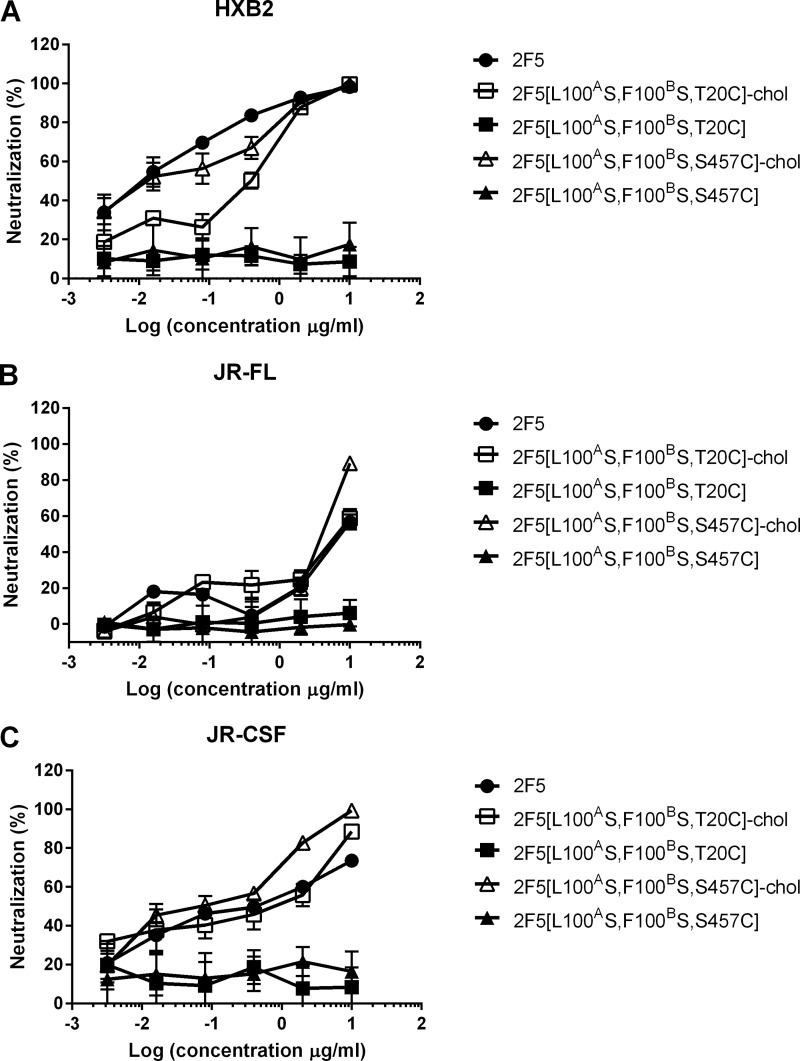
**Cholesterol conjugation rescues the antiviral activity of CDRH3-mutated 2F5.** Shown is antiviral activity of 2F5 (●), 2F5[L100^A^S,F100^B^S,T20C] (■), 2F5[L100^A^S,F100^B^S,S457C] (▴), 2F5[L100^A^S,F100^B^S,T20C]-chol (□), and 2F5[L100^A^S,F100^B^S,S457C]-chol (▵) on HIV-1 strains HXB2 (*A*), JR-FL (*B*), and JR-CSF (*C*). Antibody preparations were incubated at the indicated concentrations with pseudotype virus before infecting TZM-bl cells. Infection was determined after 72 h by reading luminescence. *Error bars*, S.D.

Our initial choice fell on V_L_ Thr^20^, which we mutated into cysteine, because modeling suggested that it might enable oriented, concomitant binding of cholesterol to the membrane and the 2F5 paratope to gp41. In addition to the Thr^20^ → Cys substitution, we introduced the two mutations Leu^100A^ → Ser and Phe^100B^ → Ser (Kabat numbering), which completely abolish the ability of 2F5 to bind lipid bilayers and its antiviral activity (5). The cholesterol-conjugated antibody 2F5[L100^A^S,F100^B^S,T20C]-chol was prepared by reaction of maleimido-PEG_12_-cholesterol with 2F5[L100^A^S,F100^B^S,T20C] according to the general scheme shown in Fig. 1. A schematic representation of this antibody together with the others discussed in the paper is given in Fig. 2. The analytical data for the antibody are shown in Fig. 3. After ensuring that 2F5[L100^A^S,F100^B^S,T20C]-chol retained binding to its peptide epitope (Fig. 4*A*), we tested it for antiviral activity on the highly sensitive HXB2 and the more resistant JR-FL and JR-CSF strains (Fig. 5). Cholesterol conjugation was able to completely rescue the antiviral activity abolished by the L100^A^S and F100^B^S mutations; on all three strains tested, 2F5[L100^A^S,F100^B^S,T20C]-chol was as potent as 2F5, whereas the control antibodies 2F5[L100^A^S,F100^B^S] and 2F5[L100^A^S,F100^B^S,T20C] were, as expected, inactive.

The choice of Thr^20^ was a conservative one, motivated by the suggestion that oriented binding is important for the mechanics of the 2F5 CDRH3 (5, 12–13), a feature that is not required by the proposed two-step mechanism (5, 6). To explore cholesterol conjugation at a distant position from the antibody paratope, we chose Ser^457^ in the C_H_3 domain of the heavy chain (Fig. 2*A*). 2F5[L100^A^S,F100^B^S,S457C]-chol was prepared by the same procedure used for 2F5[L100^A^S,F100^B^S,T20C] (Fig. 3). 2F5[L100^A^S,F100^B^S,S457C]-chol maintained binding to the peptide epitope (Fig. 4*A*). When tested for antiviral activity (Fig. 5), it was only slightly less potent than 2F5[L100^A^S,F100^B^S,T20C]-chol and 2F5. The control unconjugated antibody 2F5[L100^A^S,F100^B^S,S457C] was, as expected, inactive.

**FIGURE 6.**
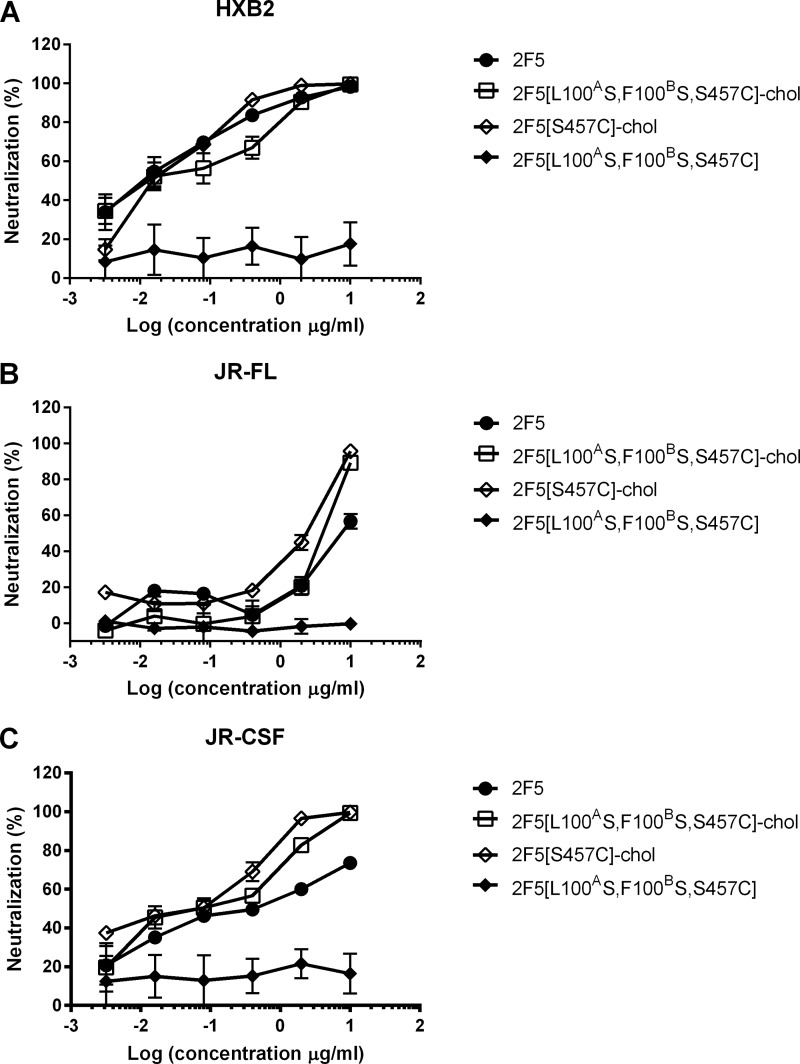
**Cholesterol conjugation potentiates the antiviral activity of WT 2F5.** Shown is antiviral activity of 2F5 (●), 2F5[L100^A^S,F100^B^S,S457C] (♦), 2F5[L100^A^S,F100^B^S,S457C]-chol (□), and 2F5[S457C]-chol (♢) on HIV-1 strains HXB2 (*A*), JR-FL (*B*), and JR-CSF (*C*). *Error bars*, S.D.

We next asked the question of whether cholesterol conjugation can synergize with the action of the CDRH3. If the 2F5 CDRH3 does indeed participate in an “extended paratope” (12, 13), selection must have optimized its properties along the entire reaction coordinate rather than simply maximizing its membrane binding capacity. It would follow that an additional membrane-binding component might add to antiviral activity. To address this question, we compared the antiviral potency of 2F5; 2F5[L100^A^S,F100^B^S,S457C]-chol, featuring cholesterol and a mutated CDRH3; and 2F5[S457C]-chol, featuring cholesterol and a WT CDRH3 (Fig. 2*A*). When tested against the HIV strain JR-FL, 2F5[S457C]-chol showed dramatically increased potency *versus* 2F5 and the control antibody 2F5[L100^A^S,F100^B^S,S457C] (Fig. 6).

Our results suggest that the antiviral activity of 2F5 (and probably 4E10) requires both dual-binding affinity and “extended paratope”; such a complex mechanism would justify the need for extensive somatic affinity maturation during an extended period of antigen exposure (37). More importantly, these results show that cholesterol conjugation can replace and actually improve upon the natural membrane-binding function of the 2F5 CDRH3.

##### Cholesterol Conjugation Potentiates the Non-membrane Binding HIV Antibody D5

The finding that cholesterol conjugation can provide additional binding energy to 2F5, without conflict with its special binding mechanism, suggests that it should work for other neutralizing antibodies. Therefore, we explored cholesterol conjugation of the HIV nAb D5, whose mechanism of action is well described and does not entail affinity for the viral or cell membrane (38). D5 binds to a highly conserved hydrophobic pocket in the N-heptad repeat region of gp41, which is critical for the assembly of the postfusion 6-helix bundle structure (39). It neutralizes a diverse range of HIV isolates but is considerably less potent than 2F5. D5 therefore represented an ideal case to test whether cholesterol conjugation would provide a more potent antiviral.

Also in this case, the V_L_ position Thr^20^ and the C_H_3 position Ser^444^ appeared suitable for cholesterol derivatization. D5[T20C]-chol and D5[S444C]-chol were prepared by the same procedure used for the cholesterol-conjugated 2F5 antibodies (Figs. 2*B* and 3).

**FIGURE 7.**
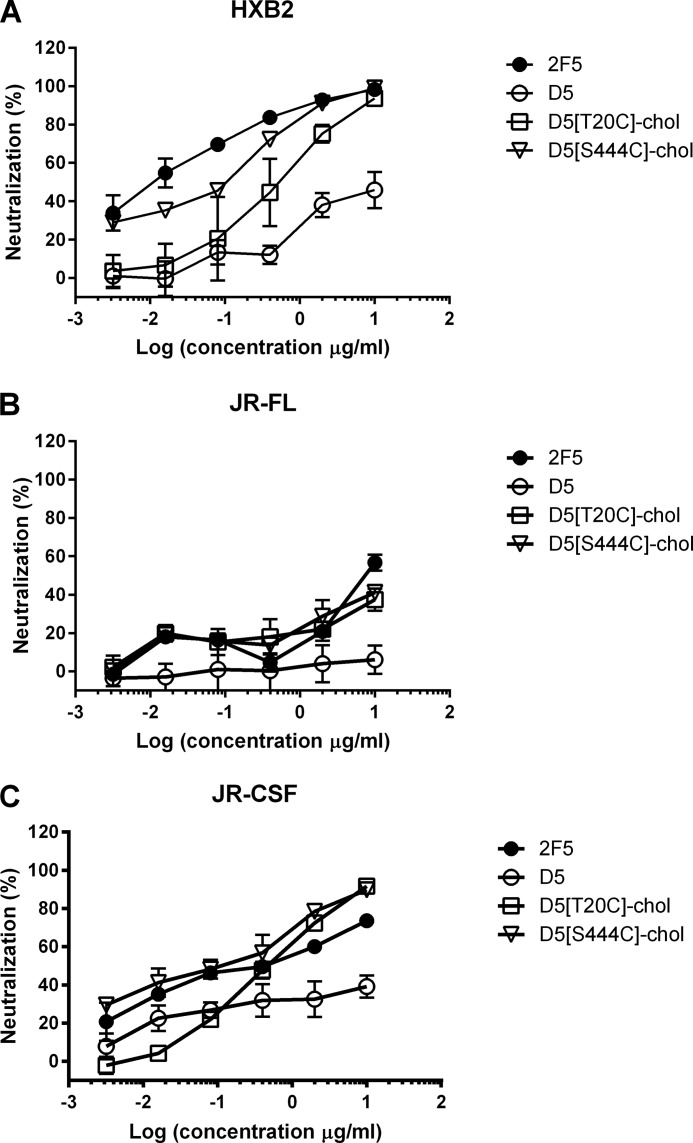
**Cholesterol conjugation potentiates the antiviral activity of antibody D5.** Shown are neutralization curves of D5 (○), D5[T20C]-chol and D5[S444C]-chol (▿), and 2F5 (●) on HIV-1 strains HXB2 (*A*), JR-FL (*B*), and JR-CSF (*C*). *Error bars*, S.D.

Like the 2F5 antibodies, D5[T20C]-chol and D5[S444C]-chol maintained binding to their peptide epitope (Fig. 4*B*). Both cholesterol-conjugated D5 antibodies were able to inhibit all three HIV strains, whereas D5 was only active on HXB2 (Fig. 7). On the latter strain, D5[T20C]-chol and D5[S444C]-chol were 10-fold and 100-fold more potent, respectively, than WT D5.

The lack of activity of WT D5 on strains JR-CSF and JR-FL was expected based on our previous data (IC_50_ > 1 μm (38)), and the neutralization observed for D5[T20C]-chol and D5[S444C]-chol indicates that cholesterol conjugation may increase the breadth of neutralization of this antibody. We extended this analysis to six more HIV strains that are insensitive to D5 neutralization and found that to a different degree, they all became sensitive to the two cholesterol-conjugated antibodies (Table 1).

**TABLE 1:** **Breadth of neutralization of cholesterol-conjugated antibodies** The antibodies shown are as follows. Ab1, 2F5; Ab2, 2F5[L100^A^S,F100^B^S,T20C]-chol; Ab3, 2F5[L100^A^S,F100^B^S,S457C]-chol; Ab4, 2F5[S457C]-chol; Ab5, D5; Ab6, D5[T20C]-chol; Ab7, D5[S444C]-chol.

Strain*^a^*	IC_50_ of pseudotyped HIV strains						
	Ab1	Ab2	Ab3	Ab4	Ab5	Ab6	Ab7
	μ*g/ml*						
HXB2	0.01	0.19	0.02	0.02	10.83	0.49	0.05
JR-FL	8.42	7.86	3.78	1.87	>100	>100	46.78
JR-CSF	0.27	0.20	0.06	0.03	>100	0.49	0.07
E21LnD43	4.48	10.01	1.36	1.06	>100	6.80	0.97
E21LnD17	20.47	21.98	2.35	0.54	>100	7.89	1.24
E21BrD9	9.67	5.56	1.72	1.33	>100	7.52	1.87
E21BrD82	19.71	15.30	2.92	0.76	>100	41.12	16.9
Km34BIR70	>100	>100	4.87	0.86	>100	11.99	15.4
Km34SeR33	>100	44.49	1.82	0.17	>100	23.51	2.70

*^a^* Characterized recombinant gp160 used in the pseudotype infectivity assay; for full phenotypic characterization, see Ref. 53. E21LnD17, clade B, coreceptor CCR5; E21LnD43, clade B, coreceptor CXCR4; E21BrD9, clade B, coreceptor CCR5; E21BrD82, clade B, coreceptor CCR5, macrophage-tropic; Km34BIR70, clade U (*env* gene not corresponding to any described group M subtype), coreceptor CCR5; Km34SeR33, clade U, coreceptor CCR5, macrophage-tropic.

In the same experiment, we also compared conjugated and unconjugated 2F5 (Table 1). These data confirm that conjugation can rescue (in position Thr^20^) and actually potentiate (in position Ser^457^) the antiviral activity of the inactive 2F5[L100^A^S,F100^B^S] mutant and can work in concert with the natural membrane-binding CDRH3 to produce a superpotent antibody (80–100% inhibition at 2 μg/ml for 5 of 6 strains).

Notably, D5[T20C]-chol showed potency comparable with that of WT 2F5 on all of the strains tested (Table 1). We may conclude that cholesterol conjugation can evolve a weak nAb with limited breadth of neutralization (38) into a potent bnAb on a par with 2F5.

##### Cholesterol-conjugated Antibodies Bind to Human Cells

**FIGURE 8.**
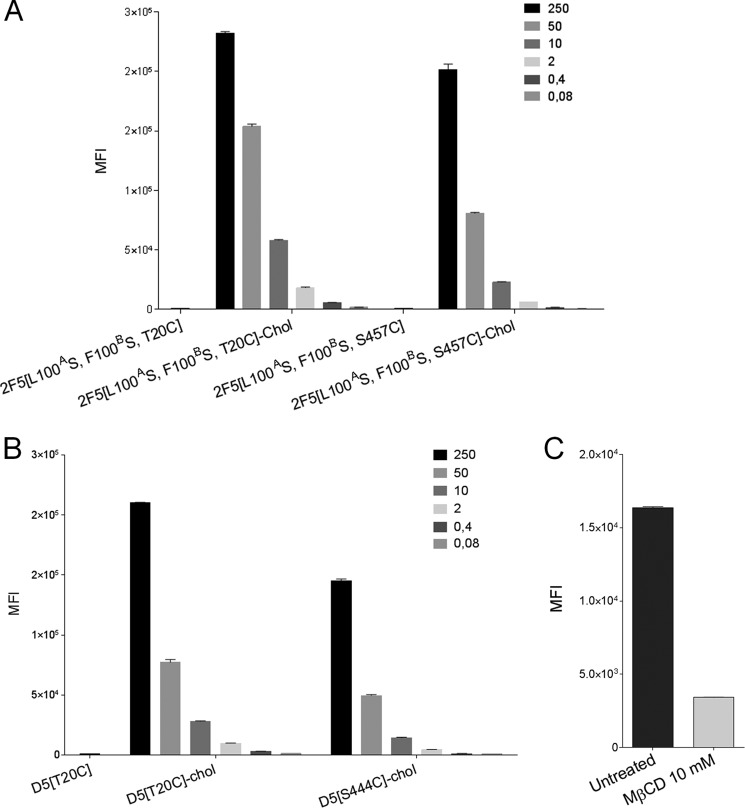
**Cholesterol conjugation provides affinity for cholesterol-rich cell membranes.** Binding of cholesterol-conjugated antibodies to HEK 293 cells, measured by flow cytometry. Values on the *vertical axis* are mean fluorescence intensities (*MFI*), and the concentrations of mAbs (nm) used in the experiment are shown in the *top right corner* of each *panel. A*, binding of 2F5, 2F5[L100^A^S,F100^B^S,T20C], 2F5[L100^A^S,F100^B^S,S457C], 2F5[L100^A^S,F100^B^S,T20C]-chol, and 2F5[L100^A^S,F100^B^S,S457C]-chol to HEK 293 cells. *B*, binding of D5, D5[T20C]-chol, and D5[S444C]-chol to HEK 293 cells. *C*, binding of 10 nm 2F5[L100^A^S,F100^B^S,T20C]-chol to HEK 293 cells is reduced by pretreatment with methyl-β-cyclodextrin (*M*β*CD*). *Error bars*, S.D.

To probe the mechanistic basis of cholesterol conjugation, we tested binding of cholesterol-conjugated antibodies to HEK 293 cells. We had previously shown that the cholesterol-conjugated antiviral peptides bind to and concentrate on the cell surface (21). Likewise, cholesterol conjugation endows both 2F5 and D5 antibodies with high affinity for human cells (Fig. 8, *A* and *B*). Binding is strongly reduced by cell treatment with methyl-β-cyclodextrin, which depletes cholesterol and leads to disorganization of lipid raft microdomains (40) (Fig. 8*C*).

**FIGURE 9.**
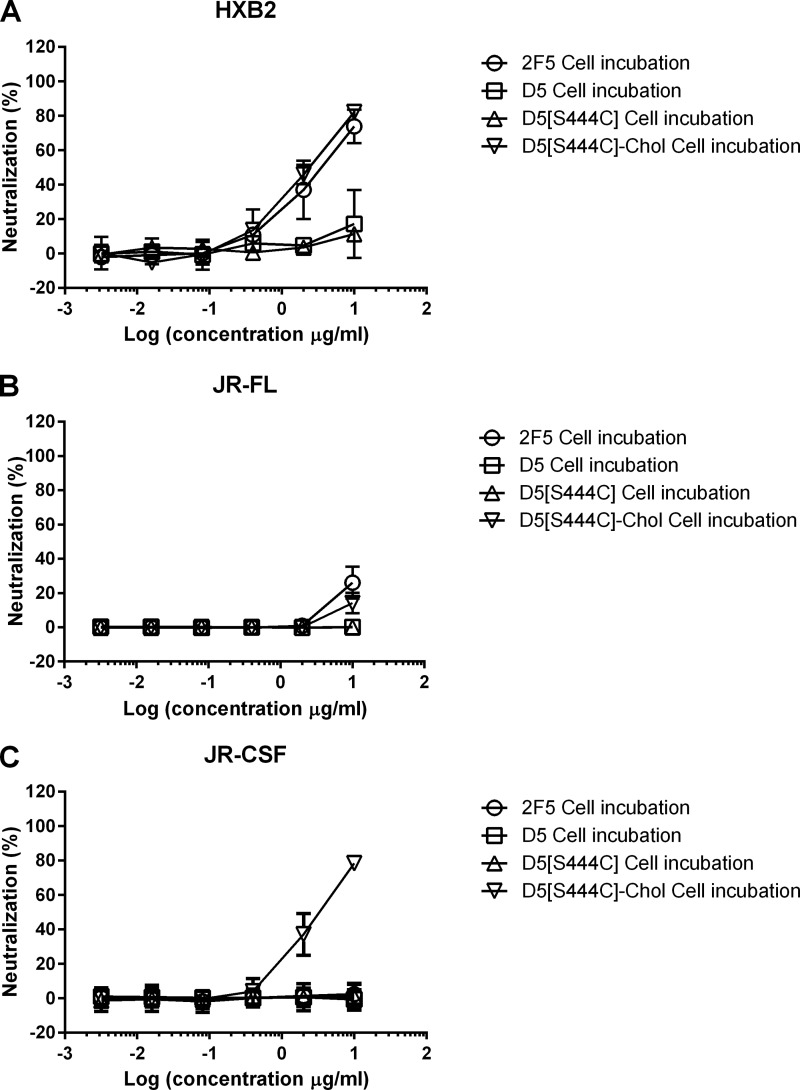
**Binding of cholesterol-conjugated antibodies is stable to cell washing.** Shown are neutralization curves of 2F5 (○), D5 (□), D5[S444C] (▵), and D5[S444C]-chol (▿) on HIV-1 strains HXB2 (*A*), JR-FL (*B*), and JR-CSF (*C*). Antibody preparations were incubated at the indicated concentrations with TZM-bl cells for 1 h. Cells were washed thoroughly with PBS, and pseudotype virus was added to cells. Infection was determined after 72 h by reading luminescence. Data are presented as a percentage of uninhibited control. All reactions were performed in triplicate. *Error bars*, S.D.

Importantly, as observed for the cholesterol-conjugated peptides (21), binding of cholesterol-conjugated D5 is stable to cell wash prior to virus addition (Fig. 9). Interestingly, the lipid raft-binding CDRH3 of WT 2F5 also provides this property, albeit to a lower degree than cholesterol conjugation, as judged by the residual ability of 2F5 to neutralize strains HXB2 and JR-FL after cell wash (Fig. 9).

##### Synergy between Cholesterol-conjugated HIV Antibodies

**FIGURE 10.**
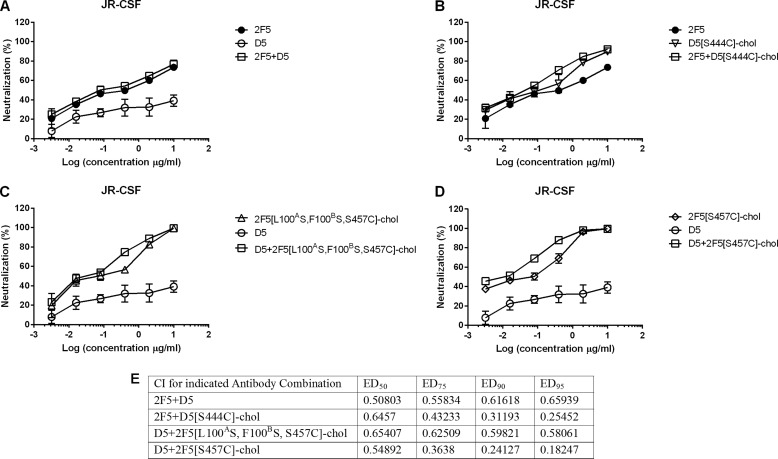
**Effect of mAb combinations in neutralizing JR-CSF.** *A*, neutralization curves of 2F5 (●), D5 (○), and 2F5 + D5 (□) at constant ratios. *B*, neutralization curves of 2F5 (●), D5[S444C]-chol (▿), and 2F5 + D5[S444C]-chol (□) at constant ratios. *C*, neutralization curves of D5 (○), 2F5[L100^A^S,F100^B^S,S457C]-chol (▵), and D5 + 2F5[L100^A^S,F100^B^S,S457C]-chol (□) at constant ratios. *D*, D5 (○), 2F5[S457C]-chol (♢), and D5 + 2F5[S457C]-chol (□). Antibody preparations were incubated at the indicated concentrations with pseudotype virus before infecting TZM-bl cells. Infection was determined after 72 h by reading luminescence. Data are presented as a percentage of uninhibited control. All reactions were performed in triplicate. *E*, CI values for combinations of 2F5 and D5 with D5[S444C]-chol, 2F5[L100^A^S,F100^B^S,S457C]-chol, or 2F5[S457C]-chol at ED_50_, ED_75_, ED_90_, and EC_95_, as computed by CompuSyn. For each set of analyses, the linear correlation coefficient *r* was >0.88, indicating a good fit to the plots (data not shown). A CI of <1 is synergism, and a CI of >1 is antagonism. *Error bars*, S.D.

The combination of 2F5 and D5 is known to be synergistic (41), and we asked if this synergy is maintained for the corresponding cholesterol-conjugated antibodies. We tested a number of combinations of unconjugated and cholesterol-conjugated 2F5 and D5 on HIV strain JR-CSF and observed synergy in all cases (Fig. 10). The most notable finding was that synergy of the naturally membrane-binding bnAb 2F5 with the non-membrane binding nAb D5 was *increased* when the latter was made membrane-binding via cholesterol conjugation. We may conclude that cholesterol conjugation, which independently potentiates each antibody, also potentiates their combination.

## DISCUSSION

We have shown here that the site-specific addition of cholesterol to an HIV antibody endows it with affinity for the lipid raft domain of the cell membrane and, because of this enrichment, potentiates the antibody-neutralizing activity. The data for D5 in particular illustrate the advantage of cholesterol conjugation because an antibody with limited potency and breadth becomes similar to the bnAb 2F5. This enhancement is independent of the specific epitope and therefore represents a way to generalize the agreed upon mechanism of binding of the HIV bnAbs 2F5 and 4E10, where the lipid binding sequence is present in the long CDRH3 of their paratope (37). This mechanism seems valid also for the MPER-binding antibody 10E8, initially described as an exception because of its exceptional neutralization potency without intrinsic affinity for membranes (42) but later shown to actually bind lipid bilayers through two hydrophobic residues in its CDRH3. In the latter work, Chen *et al.* (43) hypothesized that the superior neutralization potency of 10E8 *versus* 4E10 is related to its *differential* affinity for cholesterol-rich domains and membranes in general; such a mechanism would resemble the lipid raft-targeting effect of cholesterol conjugation.

Our findings may be relevant to the proposed role of polyreactivity, the ability of an antibody to recognize more than one unrelated antigen, in the natural response to HIV. It has been argued that for HIV, which displays its trimeric gp140 envelope spikes at very low density (44–46), monospecific bivalent binding is not possible (47). A polyreactive antibody, with both high affinity binding to a virus-specific site and low affinity binding to a secondary site on the virion and/or the target cell, may engage in bispecific bivalent interaction (also called heteroligation). 2F5 and 4E10 would then represent special cases of polyreactivity. Accordingly, the majority of HIV antibodies with high neutralizing activity isolated from memory B cells of HIV-infected patients were found to be polyreactive (47).

**FIGURE 11.**
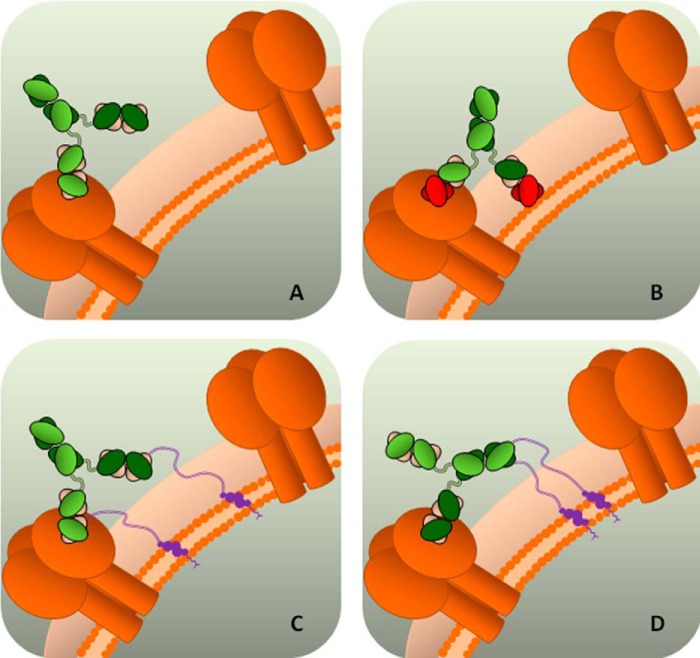
**Schematic representation of engineered polyreactivity.** *A*, a monoreactive antibody cannot display homotypic bivalent binding for the low density of target epitopes on the pathogen surface. Pathogen protein is shown in *dark orange*, the heavy chain of mAb is shown in *shades* of *green*, and light chain is shown in *pink. B*, a polyreactive antibody can display heterotypic bivalent binding through heteroligation of the protein epitope and the pathogen membrane. The polyreactive paratope is highlighted in *red. C* and *D*, engineering polyreactivity in a monospecific antibody. Shown is heteroligation of protein epitope and pathogen membrane through the cholesterol anchor (*violet*) conjugated to the variable (*C*) or constant (*D*) domain of the antibody.

Our data support this hypothesis by showing that when a non-membrane-binding antibody (D5) is made polyreactive, its antiviral potency increases. An illustration of this “engineered polyreactivity” via cholesterol conjugation is given in Fig. 11. Additionally, we found that there is increased synergism between the two cholesterol-conjugated, polyreactive antibodies 2F5 and D5 *versus* their natural variants, suggesting that a polyclonal polyreactive response, as naturally found in HIV-infected patients (47), may be particularly effective.

The scope of cholesterol conjugation remains to be established for antibodies to viruses other than HIV and even for other HIV antibodies. Although one would expect the magnitude of the effect to be maximal for fusion-inhibitory antibodies like 2F5/4E10, the preconcentration effect from additional membrane affinity might also benefit receptor-binding nAbs like b12 (which binds to the CD4-binding site) or PG9 (which binds the membrane-distal apex of the spike). It appears likely that the optimal conjugation position and linker length will be antibody-specific, although the possibility of engineering the constant region may attenuate the need for case-by-case optimization.

On the practical side, cholesterol conjugation may find application in the development of antibody therapeutics. Increased *in vitro* potency has been shown to correlate with *in vivo* effectiveness (48) and is usually achieved via affinity maturation or structure-based design (48). These approaches typically improve the *k*_off_ component of *K_D_* and may work in concert with cholesterol conjugation, which improves the *k*_on_ component. The success of cholesterol conjugation with D5 is encouraging because, in this case, affinity maturation failed to identify variants more potent than the parental nAb (49).

Cholesterol may represent a hapten and elicit the formation of anti-drug antibodies, which would limit the efficacy of chronic (but not acute) administration of a cholesterol-conjugated antibody. However, the clinical experience so far with antibody-drug conjugates (50) has not identified anti-drug antibodies as a major issue.

Finally, the advantage of cholesterol conjugation might extend beyond antiviral antibodies. Lipid rafts are a general membrane-organizing principle, and several receptors have been described to concentrate in the “raft phase” (51). Accordingly, we showed that conjugation of cholesterol to the peptide hormone oxyntomodulin, a co-agonist of the GLP-1 and glucagon receptors with antidiabetic and anti-obesity activity, produced a substantial increase in biological potency (18, 52).

## Footnotes

3 The abbreviations used are:

bnAb: broadly neutralizing antibody
nAb: neutralizing antibody
MPER: membrane-proximal external region
HEK: human embryonic kidney
CI: combination index.
